# Poly(l-γ-glutamylglutamine) Polymer Enhances Doxorubicin Accumulation in Multidrug Resistant Breast Cancer Cells

**DOI:** 10.3390/molecules21060720

**Published:** 2016-06-02

**Authors:** Ting Peng, Kai Liu, Liefang Gao, Lipeng Gao, Jing Chen, Jing Wang, Yu Liu, Yiting Wang, Zhiqiang Yan, Lei Yu

**Affiliations:** 1 Institute of Biomedical Engineering and Technology, School of Chemistry and Molecular Engineering, East China Normal University, Shanghai 200062, China; pengtingcpu@163.com (T.P.); adamlilith-lilin@163.com (K.L.); gaoliefang@163.com (L.G.); glp229@163.com (L.G.); jing.chen@sibcb.ac.cn (J.C.); jwang@nbic.ecnu.edu.cn (J.W.); ytwang@nbic.ecnu.edu.cn (Y.W.); 2 Key Laboratory of Smart Drug Delivery, Ministry of Education & PLA, Department of Pharmaceutics, School of Pharmacy, Fudan University, Shanghai 201203, China; liuyu0726@foxmail.com

**Keywords:** multidrug resistance, PGG, doxorubicin, conjugate, polymer nanomedicines

## Abstract

*Background*: Drug resistance is one of the bottlenecks of cancer chemotherapy in the clinic. Polymeric nanomedicine is one of the most promising strategies for overcoming poor chemotherapy responses due to the multidrug resistance (MDR). *Methods*: In this study, a new polymer-based drug delivery system, poly (l-γ-glutamylglutamine)-doxorubicin (PGG-Dox) conjugate, was studied in both drug-induced resistant human breast cancer MDA-MB-231/MDR cells and their parent human breast cancer MDA-MB-231 cells. The effect of PGG on facilitating the growth inhibition of Dox against multidrug resistant cells were investigated by evaluating the cytotoxicity of PGG-Dox conjugate, PGG/Dox unconjugated complex and free Dox on both cells. The underlying mechanisms in resistant cells were further studied via the intracellular traffic studies. *Results*: Both conjugated and unconjugated PGG significantly increased Dox uptake, prolonged Dox retention and reduced Dox efflux in the MDA-MB-231/MDR cells. The PGG-Dox conjugate is taken up by tumor cells mainly by pinocytosis pathway, in which PGG-Dox conjugate-containing vesicles are formed and enter the cells. *Conclusions*: This study indicated that both polymer-drug conjugate and unconjugated complex are promising strategies of overcoming resistance of anti-tumor drugs.

## 1. Introduction

Even though the anticancer efficacy of most drugs is far from satisfactory, chemotherapy is still one of routine strategies in the clinical therapy of breast cancer [1,2,3]. The anticancer efficacy of chemotherapeutic drugs largely depends on the concentration accumulated in the desired cancer cells with a certain time window (retention time), which is determined by the cellular influx/efflux ratio of the certain chemotherapy drugs in the given time frame [4].

The cellular influx of chemotherapeutic drugs to cancer cells is mainly mediated by the diffusion of hydrophobic agents and endocytosis of hydrophilic agents. The efflux of chemotherapeutic agents is mediated by the drug pump induced by cellular drug exposure. Cancer cells employ natural defense mechanisms mediated by ATP-binding cassette (ABC) transporter family [5,6], especially P-glycoprotein (P-gp) [7], to become resistant to one or more therapeutic agents, which is known as multidrug resistance (MDR) [7].

Doxorubicin (Dox) is an anthracycline with detectable red fluorescence that ranks among the most effective anticancer drugs. However, its therapeutic outcome is still limited by low dose administration and cellular drug efflux [8,9].

Polymer-based drug delivery systems (DDS) represent an extensively investigated strategy used to prolong drug exposure period, increase drug transport, achieve higher drug doses with lower toxicity and overcome the MDR mediated by P-gp [10,11]. Polymeric nanomedicines are able to enhance delivery efficacy via endocytosis-mediated uptake [12]. In addition, polymer-drug conjugates show significant decreases in the efflux of chemotherapeutic agents in cells [13,14]. The proposed mechanism of overcoming drug resistance by a polymeric pro-drug was that these polymer-drug conjugates are likely too large to be handled by ABC transporters and are consequently “trapped” within the cancer cells [15].

Various hydrophilic polymeric conjugations have been explored, including polyglutamate-paclitaxel (Ptx) [16], HPMA copolymer-Dox [17,18], HPMA copolymer-Ptx [19,20], Dextran-Dox [21,22] and poly(l-γ-glutamylglutamine) (PGG) based nanomedicines. Most recently, poly-(l-γ-glutamylglutamine)-paclitaxel nanoconjugate (PGG-Ptx) [23] demonstrated efficacious antitumor activity *in vivo* and outperformed albumin-bound Ptx nanoparticles in murine models [24]. PGG-Ptx showed prolonged half-life of total, extractable, and active free Ptx in both the plasma and tumor compartments compared with the Cremophor EL-ethanol formulation of Ptx in BABL/c nude mice bearing lung cancer NCI-H460 xenograft [25]. In addition, a free Ptx-loaded PGG-Ptx conjugate nanoparticle drug delivery system [26] and PGG-docetaxel conjugate pro-drug [27] also showed good pharmacokinetic behavior. However, the anticancer efficacy of PGG based nanomedicine in MDR cancer cells is still unknown.

The goal of this study was to investigate the therapeutic outcome of a PGG-based polymer nanomedicine in drug resistant breast cancer cells and the possible mechanism of action. We report here the increased accumulation of PGG based polymeric Dox (both conjugated and unconjugated) in resistant cancer cells, which have important implications for the design of polymer-drug conjugates for overcoming MDR. This is the first time to report that the polymer/drug complex without chemical conjugation could also help keeping the drug in the cells from drug efflux in MDR cells. This new discovery will help in the design and development of new anticancer DDS to overcome MDR for improving cancer chemotherapy in clinic.

## 2. Results 

### 2.1. Characterization of PGG-Dox

**Figure 1:**
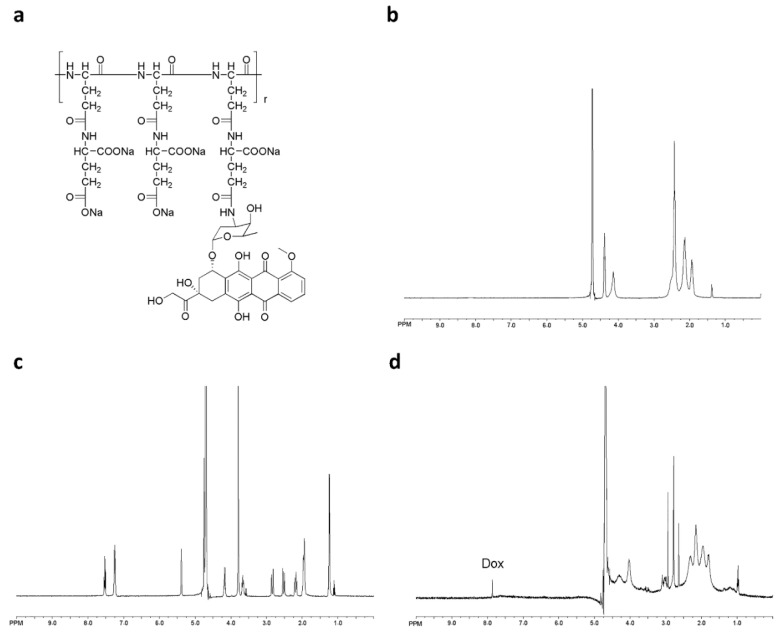
The chemical structure of PGG-Dox conjugate (**a**); ^1^H-NMR spectra of PGG polymer-length chain (**b**); free Dox (**c**) and PGG-Dox (**d**) in D_2_O.

PGG-Dox conjugate was characterized by ^1^H-NMR. Peaks corresponding to both PGG and Dox conjugates are shown in Figure 1b,d, respectively. Free Dox and PGG-Dox were identified and confirmed by proton chemical shifts at 7.0–8.0 ppm for its aromatic protons (Figure 1c,d). The PGG-Dox showed a drug loading capacity of 35% as calculated according to our previous reports [23].

**Figure 2:**
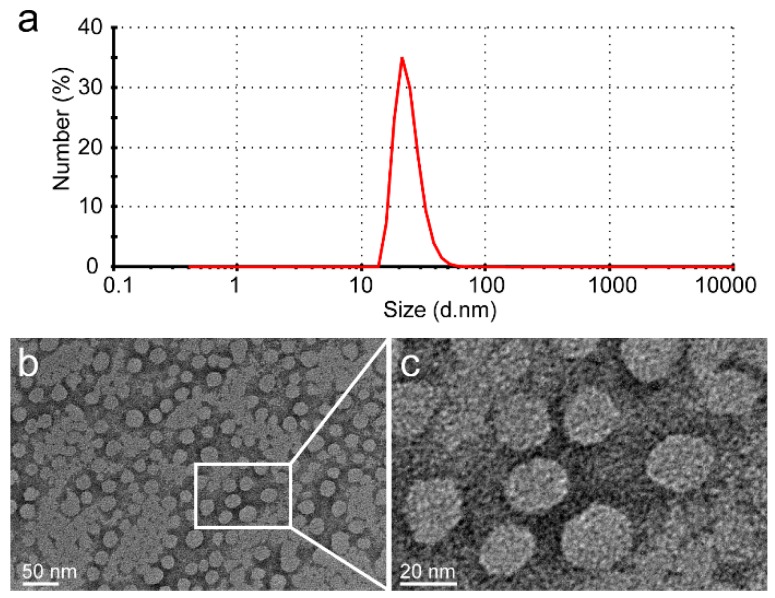
The DLS results of PGG-Dox nanoparticles showed that the average particle size is 20 nm, and that PDI is 0.36 (**a**); The PGG-Dox nanoparticles exhibited uniform spherical morphology as shown in the TEM images (**b**,**c**).

The glutamic acid linker was able to provide additional water-solubility so that the polymer could be loaded to a high level with Dox, while having sufficient flexibility. The Dox moieties could form the hydrophobic inner core of nanoparticles, and the PGG polymer forms the hydrophilic shell. The DLS results show that the mean size of PGG-Dox nanoparticles was about 20 nm, and the PDI was 0.36 (Figure 2). The TEM images of showed that PGG-Dox nanoparticles have a uniform spherical morphology, with a particle size of around 25 nm.

### 2.2. Evaluation of MDA-MB-231/MDR

**Figure 3:**
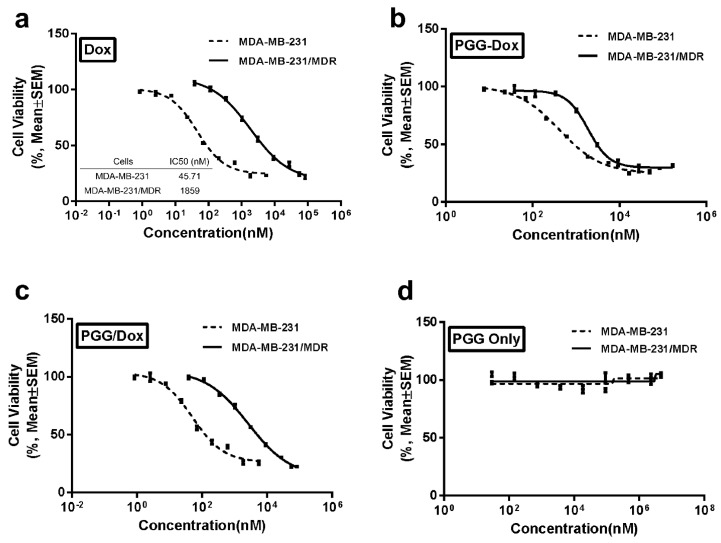
Inhibitory effects of Dox (**a**); PGG-Dox (**b**); PGG-Dox (**c**) and PGG polymers (**d**) on the proliferation of MDA-MB-231 and MDA-MB-231/MDR cell lines measured by MTT assay. Data are presented as the mean ± S.D. of three independent experiments (*n* = 3) with triplicate (*n* = 3) measurements for each experiment (*p* < 0.05).

In order to mimic MDR occurring in clinical trials, MDA-MB-231 cells were selectively induced with Dox in a stepwise manner. MTT assays were performed to evaluate the resistance of selected cell line. Figure 3a reports the significantly different cell viability between the wild-type cells and the resistant cells. Compared with wild-type cells, the resistant cell line showed 40-fold increased IC_50_ values, which indicated sufficient resistance of the induced cell lines.

**Figure 4:**
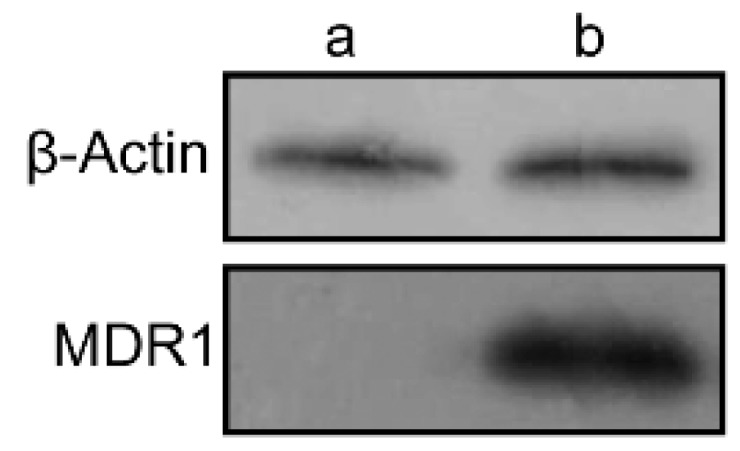
The expression of MDR1 protein in MDA-MB-231 (**a**) and MDA-MB-231/MDR cells (**b**) by western blotting assay.

Western blotting assays were carried out to confirm the expression of P-gp in wild and resistant cells. There is almost no expression of P-gp in wild MDA-MB-231 cells, whereas in MDA-MB-231/MDR cells, significantly P-gp protein expression were detected (Figure 4). The P-gp protein may account for the efflux of anti-tumor agents in MDR cells, thereby enhancing the survival rate of cells uncer high concentration of Dox (Figure 3a).

### 2.3. Antitumor Effect of PGG Based Nanomedicine in MDA-MB-231/MDR

Both wild-type and resistant cells were incubated with PGG-Dox to determine the anticancer effect of the PGG-based nanomedicine. A clear dose-dependent cytotoxicity was seen on both cell lines as shown in Figure 3b. The IC_50_ value for free Dox on MDA-MB-231/MDR cell line showed a 40-fold increase compared with MDA-MB-231 cells (Figure 3a), whereas the multiples for PGG-Dox and PGG/Dox were 3.60 and 35.6, respectively (converted to equivalent Dox concentration, Figure 3b,c). No obvious toxicity of PGG polymers was found (Figure 3d). These results indicated that conjugated PGG can reduce Dox resistance in MDA-MB-231/MDR cells.

### 2.4. Effect of PGG on Drug Accumulation in MDA-MB-231/MDR

**Figure 5:**
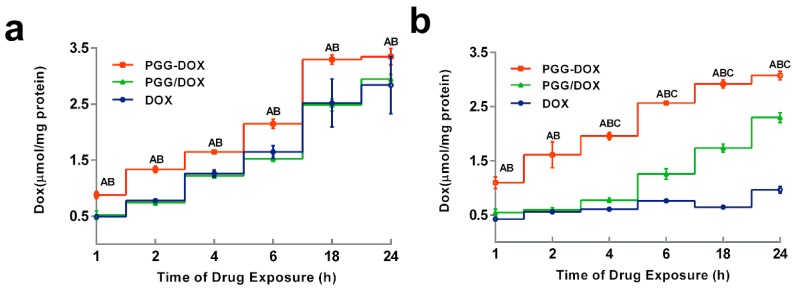
Dynamic accumulation of PGG-Dox, PGG/Dox and Dox in MDA-MB-231 (**a**) and MDA-MB-231/MDR (**b**). Results were normalized with cellular protein level and presented as the mean ± S.E.M. of three independent experiments (*n* = 3) measurements for each experiment (A: PGG-Dox *vs.* PGG/Dox, *p* < 0.05; B, PGG-Dox *vs.* Dox, *p* < 0.05; C: PGG/Dox *vs.* Dox, *p* < 0.05).

**Figure 6:**
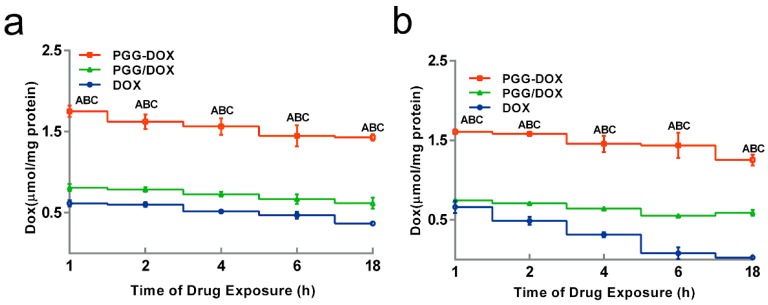
Effect of PGG-Dox, PGG/Dox and Dox on drug retention in MDA-MB-231 (**a**) and MDA-MB-231/MDR (**b**). Results were normalized with cellular protein level and presented as the mean ± SEM of three independent experiments (*n* = 3) measurements for each experiment (A: PGG-Dox *vs.* PGG/Dox, *p* < 0.05; B: PGG-Dox *vs.* Dox, *p* < 0.05; C: PGG/Dox *vs.* Dox, *p* < 0.05).

To explain the inhibitory effect of PGG-Dox on MDA-MB-231/MDR cells, the cellular accumulation and retention of Dox was measured, and the results were shown in Figure 5 and Figure 6. MDA-MB-231/MDR accumulated 57% less Dox at 24 h than wild-type cells when exposed to free Dox (purple line in Figure 5a,b), while the difference is insignificant when exposed to PGG-Dox (red line in Figure 5a,b). Further, the total accumulation of PGG-Dox was 17% higher than that of free Dox in wild-type cells at 24 h (Figure 5a), comparing with 217% in resistant cells (Figure 5b). Figure 6 indicates the decline of intracellular Dox concentration caused by drug efflux within 18 h. The cellular efflux of Dox was faster in resistant cells than in wild-type when treated with free Dox. There is almost no free DOX in MDA-MB-231/MDR cells after 18 h. MDA-MB-231/MDR pre-treated with PGG-Dox (Figure 6b) showed enhanced cellular Dox retention, there was nearly 6-fold Dox retention in resistant cells at 18 h.

**Figure 7:**
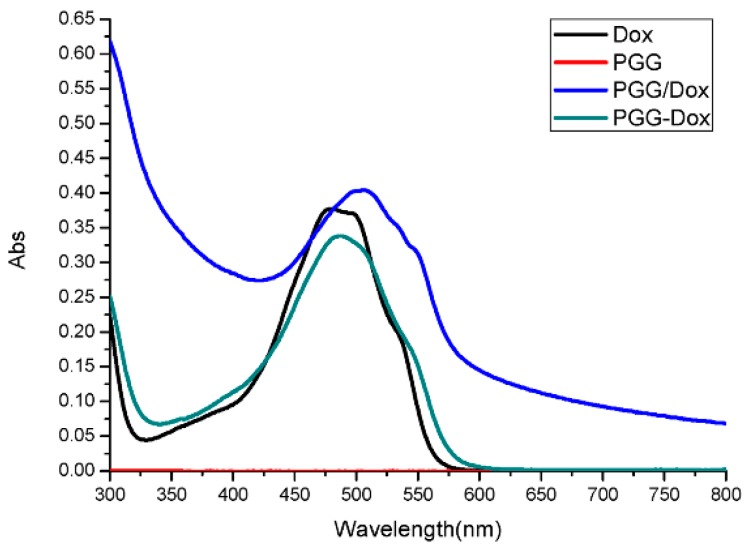
UV-vis absorption spectra of Dox, PGG, PGG/Dox and PGG-Dox (Abs, absorption).

To our surprise, though PGG/Dox complex did not perform as well as PGG-Dox in MDA-MB-231/MDR, resistant cells treated with PGG/Dox complex as more Dox was accumulated than those treated with free Dox after a 4–6 h period (Figure 5b). In contrast, no similar phenomenon was observed in wild-type cells (Figure 5a). In addition, enhanced Dox retention in MDA-MB-231/MDR treated with PGG/Dox complex was initiated since the very beginning (Figure 6b). A UV-visible spectroscopy study was performed to address this phenomenon. A clear red shift indicating interaction between PGG and free Dox was seen in the UV-vis absorption spectra of PGG/Dox after 4 h (Figure 7). Therefore, the enhanced accumulation of Dox may be attributed (at least partly) to its interaction with the PGG polymer.

Then, intracellular Dox uptake was calculated. As shown in Table 1, PGG conjugation greatly increased Dox uptake by resistant cells and also contributed to Dox retention. A clear time-dependent intracellular uptake profile was seen in PGG-Dox treated cells. The similar pattern was only seen in cells treated with PGG/Dox after 4 h. The results indicated that increasing the influx and decreasing the efflux are two possible mechanism of the enhancement of Dox accumulation in resistant cells.

### 2.5. Effect of PGG on Dox Uptake Pathway in MDA-MB-231/MDR

**Figure 8:**
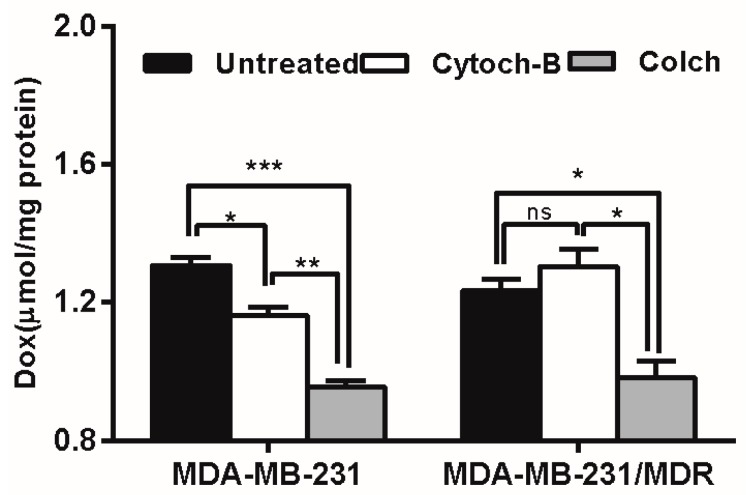
Effect of endocytosis inhibitors pretreatment on PGG-Dox accumulation in MDA-MB-231/MDR. Results were normalized with cellular protein level and presented as the Mean ± SEM of three independent experiments (*n* = 3) measurements for each experiment (* *p* < 0.05; ** *p* < 0.01; *** *p* < 0.005; ns, not significant; Colch, colchicine; Cytoch-B, cytochalasin-B).

The intracellular uptake of polymer-drug conjugates is mainly mediated by endocytosis, including pinocytosis (taking up fluids and solutes) and phagocytosis (taking up large particles) [28]. To determine which form of endocytosis is mainly involved in the elevating drug uptake in MDR cells, an endocytosis inhibition study was conducted. According to Figure 8, cytochalasin-B caused moderate inhibition of Dox accumulation in wild-type cells, but enhanced Dox accumulation (though not significantly) in resistant cells, whereas pretreatment with colchicine caused severe inhibition of Dox accumulation in both wild type and resistant cell lines. Since colchicine is a specific inhibitor of pinocytosis, the result suggested that the pinocytosis may be mainly responsible for the uptake of Dox delivered by PGG-Dox.

**Figure 9:**
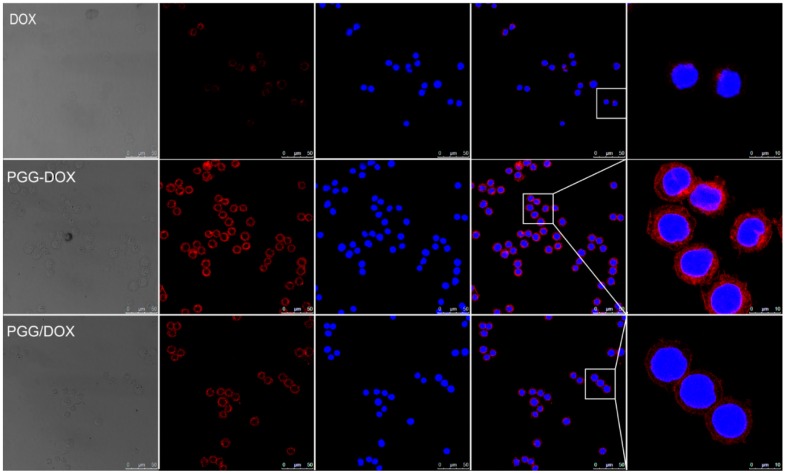
Fluorescent microscope images demonstrating the internalization of Dox, PGG-Dox and PGG/Dox by MDA-MB-231/MDR cells following 2 h incubation.

Confocal laser scanning microscopy (CLSM) was applied to confirm the internalization of free Dox, PGG-Dox and PGG/Dox in MDA-MB-231/MDR cells. In Figure 9, the nucleus was visualized as the blue fluorescence after staining cells with DAPI, while Dox or PGG-Dox were shown as red fluorescence. Compared with free Dox, significantly increased cellular uptake of PGG-Dox was observed. The cellular uptake of PGG/Dox is also slightly increased compared with that of free Dox. The increased drug retention should be resulted from the decreased drug efflux as evidenced above.

## 3. Discussion

Drug resistance is so far still one of the major hurdles of successful chemotherapy in the clinic. The basic principle of drug resistance is that there are not enough therapeutic agents in the desired cells. The amount and concentration of the therapeutic agent accumulation in the desired cancer cells are determined by the net flux values of the therapeutic agents, which are determined by the influx rate and efflux rate. Thus, the drug resistance can be overcome by increasing the drug influx rate and reducing the drug efflux rate.

In the structure of PGG-Dox, the glutamic acid linker can provide additional water-solubility for the conjugate. Therefore, the PGG polymer could form a hydrophilic shell, and the Dox moieties interact with each other and form hydrophobic inner core of nanoparticles. Besides, the linkages between the monomer of PGG polymer, between the linker (glutamic acid) and PGG backbone, and between the linker and Dox are all amido bonds, like in native proteins or peptides. Therefore, the PGG-Dox could be easily degraded in lysosomes or tumor tissues and release free Dox.

The drug accumulation in resistant cell lines is a dynamic process determined by the intracellular drug influx and efflux. If the influx rate is faster than efflux and the drug concentration reaches or surpasses the therapeutic level in the desired cancer cells while maintaining a certain therapeutic time window, the drug will show good anticancer efficacy; and *vice versa*. Endocytosis is mainly responsible for the uptake of polymeric nanomedicines [29]. Our previous study reported the molecular weight of polymeric pro-drug appear to matter in the uptake scheme [30]. Nanoparticles with a diameter around 50 nm enjoyed the highest uptake rate via endocytosis [23]. Even though many mechanisms of drug resistance were proposed, including target alternation and drug metabolism [31], P-gp still serves as the prime responsible factor. Effective chemotherapy is positively correlated to reasonable drug accumulation and action time [4]. However, once P-gp protein is induced, the cancer cells will pump the antitumor agents out of the cells, resulting in failure of cancer cell killing.

We selected the human breast cancer cell lines MDA-MB-231 as an initial cell line for preparing MDA-MB-231/MDR with stepwise exposure with both doxorubicin (Dox) and paclitaxel (Ptx). The resulting human breast multidrug resistant cancer cells showed a 40-fold IC50 value of Dox.

With the multidrug resistant human breast cancer cell line (MDA-MB-231/MDR) described above, we analyzed the effect of PGG polymer on the antitumor efficacy. The antitumor effect in MDR cells should not attributed to the cytotoxicity of PGG backbone (Figure 3d). In addition to improved intracellular Dox uptake (Figure 5b), PGG-Dox conjugate resulted in substantial enhancement of Dox retention in induced MDR human breast cancer cell line (Figure 6b). The improved total accumulation of Dox resulted from enhanced drug uptake and retention may account for overcoming the MDR.

In endocytosis inhibition, because P-gp could inhibit the effects of colchicine and cytochalasin-B [32] they are both excretion substrates [33]. Various magnitudes of Dox accumulation enhancement may lead in resistant cells (Figure 8, right panel). However, PGG-Dox treated cells were still more responsive to colchicine, indicating the enhanced uptake of Dox was mainly contributed by conjugating Dox to PGG polymer to form PGG-Dox conjugate which enters cells mainly by the pinocytosis pathway. The enhanced Dox uptake mediated by PGG was verified by CLSM. A cytoplasm-retained manner in cells treated with PGG-Dox (Figure 9) illustrated considerable Dox was delivered in the form of PGG-Dox rather than nucleus-stained free Dox [34].

Interestingly, unconjugated PGG polymer significantly improved Dox uptake (Figure 5) in long time intervals (4–24 h) and Dox retention after 2 h pretreatment by free Dox solution (Figure 6). A red shift in the UV-absorption of PGG/Dox mixture for 4 h indicated that the interaction between opposite-charged free Dox and PGG polymers may be account for the enhanced Dox accumulation.

## 4. Materials and Methods

### 4.1. Materials

Poly(l-glutamate) (PGA), *N*,*N*-dimethylformamide (DMF), sodium bicarbonate, fluorescein isothiocyanate (FITC), and 4-di(methylamino)pyridine (DMAP) were purchased from Sigma-Aldrich Chemical Co, (St Louis, MO, USA). *N*-(3-Dimethylaminopropyl)-*N*′-ethylcarbodiimide (EDC), 4′-6-diamidino-2-phenylindole (DAPI) and trifluoroacetic acid were purchased from Novabiochem (La Jolla, CA, USA). 1-Hydroxybenzotriazole was purchased from Spectrum (Gardena, CA, USA). Dox and Ptx were purchased from NuBlocks (Vista, CA, USA). All the chemicals and reagents were used as received without further purification.

### 4.2. Preparation of PGG-Dox Conjugate

PGG acid was synthesized according to our previous procedures [23]. PGA (Mw 24,880 Da) was modified by adding another glutamic acid as a linker to each glutamic acid in the polymer backbone to obtain PGG acid (Mw 53,180 Da) [23]. PGG acid was then mixed with DMF in the flask and stirred for 1 h. Then, DMAP and EDC were added in the mixture solution and stirred for 15 min. Dox was added in the resulting solution and reacted for 24 h at R.T. The mixture solution was then poured into HCl solution, and stirred to form a suspension. Then, the suspension was subjected to centrifugation at 5000 rpm for 10 min, and the sediment was re-suspended with HCl solution. After discarding supernatants, the sediments were combined and dissolved in NaHCO_3_ solution. After being stirred at room temperature for 1 h, the solution was filtered, dialyzed for 48 h and concentrated by tangential flow filtration (Millipore, Billerica, MA, USA). The resulting solution was finally lyophilized to obtain PGG-Dox.

### 4.3. ^1^H-NMR Spectroscopy and Dynamic Light-Scattering Measurements

^1^H-NMR spectra were measured on a model? NMR spectrometer (Bruker, Billerica, MA, USA) using D_2_O for Dox, PGG and PGG–Dox. Chemical shifts were reported in ppm. PGG-Dox conjugate solution (2.0 mg/mL) was prepared in ultrapure water. The particle size of PGG-Dox was determined by dynamic light-scattering (DLS) using a Nano-ZS zetasizer (Malvern, Worcestershire, UK) equipped with He-Ne laser (4 mW, 633 nm) light source and 90° angle scattered-light collection configuration.

### 4.4. Cell Culture

Human breast cancer MDA-MB-231 cell line (Cell Bank of China Scientific Academy, Shanghai, China) was cultured in L-15 medium (Gibco, Grand Island, NY, USA). The medium was supplemented with 10% fetal bovine serum (BioInd, Beit Haemek, Israel), 100 U/mL penicillin and streptomycin (Invitrogen, Carlsbad, CA, USA). Cells were incubated at 37 °C in 100% air atmosphere.

### 4.5. Development of Resistant Breast Cancer Cell Line

MDA-MB-231 cells were seeded on cell culture flasks (Corning, Union City, CA, USA) in the cell medium supplemented with Ptx and Dox. The initial concentration of Ptx was 50 μmol/mL. After 48 h, the cells was washed with PBS to remove floating cells, and the culture was changed to normal cell medium without Ptx. The concentration of Ptx was increased passage after passage by 25%–50% depending on the growing status of cells. When the cells can stably live in the cell medium with Ptx, the cells was cultured with cell medium with Dox in the same manner with Ptx. Finally, the cell line MDA-MB-231/MDR was generated. Western blotting assays were carried out to confirm the expression of P-gp protein in wild cells and resistant cells.

### 4.6. In Vitro Cytotoxicity Assay

The MTT assay was performed as described previously [2] to assess the cytotoxicity of different Dox formulations, including free Dox, PGG-DOX, PGG and PGG/DOX (the physical mixture of PGG and Dox with the same proportion as PGG-Dox). Cells were seeded in 96-well plates (Corning) at 1 × 10^5^ cells/100 μL medium. Gradient dilutions of the above-mentioned Dox formulations were added to the plate and incubated for 72 h. Percent cell viability was calculated on the basis of optical density values of sample wells *versus* reference wells, and the IC_50_ was calculated by using the Prism 5.0 software (Graphpad Software, Inc., La Jolla, CA, USA).

### 4.7. In Vitro Cellular Drug Accumulation and Retention Studies

MDA-MB-231 and MDA-MB-231/MDR cells were plated onto 24-well plates and incubated overnight. Dox, PGG-Dox and PGG/Dox were added into each well. All samples were diluted with L-15 medium and adjusted to 10 μM Dox (or equivalent concentration). Drug-free medium was used as the negative control. In the cellular uptake study, the cells were washed with ice-cold PBS (pH 7.4) and lysed with PBS containing 1% Triton X-100 at predetermined time intervals after removing the supernatant. In the cellular retention study, cells were incubated with different test samples for 2 h and then, the supernatant was replaced with drug-free L-15 medium. At predetermined time intervals, cells were treated as described above. Drug concentrations in the cell lysates were measured with a microplate fluorometer (Molecular Devices, Sunnyvale, CA, USA) with 485/595 nm excitation/emission. Cellular drug concentration is expressed in nanomoles/milligram of protein. Then the uptake was calculated according to the following formula:

(a) Net Accumulation = Dox Accumulation (last time point) − Dox Accumulation (former time point)
(b) Net Efflux = Dox Retention (former time point) − Dox Retention (last time point)
(c) Net Uptake = Net Accumulation + Net Efflux

### 4.8. Endocytosis Inhibition Studies

To determine the cell uptake pathway, the drug accumulation after endocytosis inhibition was measured according to Wong *et al*. [28] with modification. Cells were seeded in 24-well plate and pretreated with 25 μg/mL pinocytosis inhibitor colchicine or 5 μg/mL phagocytosis inhibitor cytochalasin-B, in L-15 for 1 h at 37 °C. After removing the pretreatment solution, the cells were washed with pre-warmed PBS, incubated with test samples containing PGG-Dox (equivalent to 10 g/mL Dox). Cellular Dox accumulation at 2 h was measured, and the test of protein standardization and membrane integrity were performed. Dox uptakes of cells pretreated with endocytosis inhibitors are compared with those without pretreatment, and the reduction in Dox accumulation caused by the inhibitors is expressed in nanomoles/milligram of protein.

### 4.9. Transmission Electron Microscope Study

In order to observe the pathway of cellular internalization directly, both wild-type and resistant cells were incubated with PGG-Dox for 2 h. After washing three times with ice-cold PBS, cells were fixed in 4% paraformaldehyde, rinsed twice with 6.8% sucrose and post-fixed in 1% OsO_4_. Then, they were embedded in Spurr’s resin and polymerized at 70 °C overnight after dehydrating in graded alcohol series. Ultrathin sections were cut with a diamond knife and loaded onto TEM grids. The sections were examined by a JEM-2100 electron microscope (Jeol, Tokyo, Japan) at an accelerating voltage of 6 kV.

### 4.10. Confocal Laser Scanning Microscopy

MDA-MB-231/MDR (4 × 10^5^) cells were seeded in 60 mm culture dishes (Corning) incubated for 24 h. Each well contained a poly-L-lysine-coated cover slip. Following the incubation, Dox, PGG-Dox conjugate or PGG/Dox was added into each well and incubated for 2 h. After washing three times with ice cold PBS, cells were fixed in 4% paraformaldehyde, mounted with ProLong Gold Antifade Reagent with DAPI (Invitrogen). Images were taken with an Olympus Fluoview 1000 confocal microscope (Olympus, Melville, NY, USA) equipped with a 60× water objective.

### 4.11. UV-Visible Spectroscopy

To investigate the role of PGG in unconjugated Dox uptake, UV-vis absorption spectra of PGG, Dox, PGG-Dox and PGG/Dox was recorded at 4 h after dissolved into pure water. All measures were performed at room temperature using a TU-1901 UV-visible spectrophotometer (Purkinje General, Beijing, China).

### 4.12. Statistical Analysis

Values shown are representative of triplicate determinations in two or more experiments. Student’s *t*-test was used to compare the differences between groups (Graph-Pad Prism software, GraphPad Software, San Diego, CA, USA). Unless otherwise indicated, results are given as mean & SEM and results with *p* < 0.05 were considered statistically significant.

## 5. Conclusions

In summary, this study suggest that PGG polymer effectively increase the conjugated or unconjugated Dox accumulation in MDR cells. PGG polymer can be successfully employed for the delivery of antitumor agents with higher uptake and retention to overcome MDR.

## Figures and Tables

**Table 1:** (**a**) Calculated net uptake (μmol/mg protein) of Dox, PGG-Dox and PGG/Dox in resistant cells and (**b**) uptake increase rate at different time intervals.

**a**				
**Net Uptake**	**18 h–6 h**	**6 h–2 h**	**4 h–2 h**	**2 h–1 h**
Dox	−0.063	0.387	0.226	0.307
PGG-Dox	0.536	0.627	0.473	0.540
PGG/Dox	0.442	0.572	0.244	0.088
**b**				
**Increase Rate**	**18 h–6 h**	**6 h–2 h**	**4 h–2 h**	**2 h–1 h**
PGG-Dox	950%	62%	109%	76%
PGG/Dox	801%	48%	8%	−71%

## Abbreviations

MDR	Multi-drug resistance
PGG-Dox	Poly(l-γ-glutamylglutamine)-doxorubicin
R.T.	Room temperature
Dox	Doxorubicin
ABC	ATP-binding cassette
P-gp	P-glycoprotein
DDS	Drug delivery systems
Ptx	Paclitaxel
HPMA	*N*-(2-Hydroxypropyl)methacrylamide
DMFl	*N*,*N*-Dimethylformamide
FITC	Fluorescein isothiocyanate
DMAP	4-Di(methylamino)pyridine
EDC	*N*-(3-Dimethylaminopropyl)-*N*′-ethylcarbodiimide
DAPI	4′-6-Diamidino-2-phenylindole
DLS	Dynamic light-scattering
NMR	Nuclear Magnetic Resonance
TEM	Transmission electron microscopy
CLSM	Confocal laser scanning microscopy

## References

[1] Du Y., Yamaguchi H., Wei Y., Hsu J.L., Wang H.L., Hsu Y.H., Lin W.C., Yu W.H., Leonard P.G., Lee G.R.T. (2016). Blocking c-Met-mediated PARP1 phosphorylation enhances anti-tumor effects of PARP inhibitors. Nat. Med..

[2] Yan Z., Zhan C., Wen Z., Feng L., Wang F., Liu Y., Yang X., Dong Q., Liu M., Lu W. (2011). LyP-1-conjugated doxorubicin-loaded liposomes suppress lymphatic metastasis by inhibiting lymph node metastases and destroying tumor lymphatics. Nanotechnology.

[3] Nam J.S., Sharma A.R., Nguyen L.T., Chakraborty C., Sharma G., Lee S.S. (2016). Application of Bioactive Quercetin in Oncotherapy: From Nutrition to Nanomedicine. Molecules.

[4] Millenbaugh N.J., Wientjes M.G., Au J.L. (2000). A pharmacodynamic analysis method to determine the relative importance of drug concentration and treatment time on effect. Cancer Chemother. Pharmacol..

[5] Yamanaka N., Marques G., O’Connor M.B. (2015). Vesicle-Mediated Steroid Hormone Secretion in Drosophila melanogaster. Cell.

[6] Goedeke L., Rotllan N., Canfran-Duque A., Aranda J.F., Ramirez C.M., Araldi E., Lin C.S., Anderson N.N., Wagschal A., de Cabo R. (2015). MicroRNA-148a regulates LDL receptor and ABCA1 expression to control circulating lipoprotein levels. Nat. Med..

[7] Pan G., Li T., Zeng Q., Wang X., Zhu Y. (2016). Alisol F 24 Acetate Enhances Chemosensitivity and Apoptosis of MCF-7/DOX Cells by Inhibiting P-Glycoprotein-Mediated Drug Efflux. Molecules.

[8] Riganti C., Gazzano E., Gulino G.R., Volante M., Ghigo D., Kopecka J. (2015). Two repeated low doses of doxorubicin are more effective than a single high dose against tumors overexpressing P-glycoprotein. Cancer Lett..

[9] Lee Y.K., Choi J., Wang W., Lee S., Nam T.H., Choi W.S., Kim C.J., Lee J.K., Kim S.H., Kang S.S. (2013). Nullifying tumor efflux by prolonged endolysosome vesicles: Development of low dose anticancer-carbon nanotube drug. ACS Nano.

[10] Hu C.M., Fang R.H., Wang K.C., Luk B.T., Thamphiwatana S., Dehaini D., Nguyen P., Angsantikul P., Wen C.H., Kroll A.V. (2015). Nanoparticle biointerfacing by platelet membrane cloaking. Nature.

[11] Tomalova B., Sirova M., Rossmann P., Pola R., Strohalm J., Chytil P., Cerny V., Tomala J., Kabesova M., Rihova B. (2016). The structure-dependent toxicity, pharmacokinetics and anti-tumour activity of HPMA copolymer conjugates in the treatment of solid tumours and leukaemia. J. Control. Release.

[12] Duncan R., Richardson S.C. (2012). Endocytosis and intracellular trafficking as gateways for nanomedicine delivery: Opportunities and challenges. Mol. Pharm..

[13] Tu Y., Zhu L. (2015). Enhancing cancer targeting and anticancer activity by a stimulus-sensitive multifunctional polymer-drug conjugate. J. Control. Release.

[14] Bondar O.V., Badeev Y.V., Shtyrlin Y.G., Abdullin T.I. (2014). Lipid-like trifunctional block copolymers of ethylene oxide and propylene oxide: Effective and cytocompatible modulators of intracellular drug delivery. Int. J. Pharm..

[15] Roy A., Ernsting M.J., Undzys E., Li S.D. (2015). A highly tumor-targeted nanoparticle of podophyllotoxin penetrated tumor core and regressed multidrug resistant tumors. Biomaterials.

[16] Bonomi P. (2007). Paclitaxel poliglumex (PPX, CT-2103): Macromolecular medicine for advanced non-small-cell lung cancer. Expert Rev. Anticancer Ther..

[17] Duncan R., Vicent M.J. (2010). Do HPMA copolymer conjugates have a future as clinically useful nanomedicines? A critical overview of current status and future opportunities. Adv. Drug Deliv. Rev..

[18] Etrych T., Sirova M., Starovoytova L., Rihova B., Ulbrich K. (2010). HPMA copolymer conjugates of paclitaxel and docetaxel with pH-controlled drug release. Mol. Pharm..

[19] Shi Y., van der Meel R., Theek B., Oude Blenke E., Pieters E.H., Fens M.H., Ehling J., Schiffelers R.M., Storm G., van Nostrum C.F. (2015). Complete Regression of Xenograft Tumors upon Targeted Delivery of Paclitaxel via Pi-Pi Stacking Stabilized Polymeric Micelles. ACS Nano.

[20] Larson N., Yang J., Ray A., Cheney D.L., Ghandehari H., Kopecek J. (2013). Biodegradable multiblock poly(*N*-2-hydroxypropyl)methacrylamide gemcitabine and paclitaxel conjugates for ovarian cancer cell combination treatment. Int. J. Pharm..

[21] Jeong Y.I., Kim do H., Chung C.W., Yoo J.J., Choi K.H., Kim C.H., Ha S.H., Kang D.H. (2011). Doxorubicin-incorporated polymeric micelles composed of dextran-b-poly(dl-lactide-co-glycolide) copolymer. Int. J. Nanomed..

[22] Mitra S., Gaur U., Ghosh P.C., Maitra A.N. (2001). Tumour targeted delivery of encapsulated dextran-doxorubicin conjugate using chitosan nanoparticles as carrier. J. Control. Release.

[23] Van S., Das S.K., Wang X., Feng Z., Jin Y., Hou Z., Chen F., Pham A., Jiang N., Howell S.B. (2010). Synthesis, characterization, and biological evaluation of poly(l-gamma-glutamyl-glutamine)- paclitaxel nanoconjugate. Int. J. Nanomed..

[24] Feng Z., Zhao G., Yu L., Gough D., Howell S.B. (2010). Preclinical efficacy studies of a novel nanoparticle-based formulation of paclitaxel that out-performs Abraxane. Cancer Chemother. Pharmacol..

[25] Wang X., Zhao G., Van S., Jiang N., Yu L., Vera D., Howell S.B. (2010). Pharmacokinetics and tissue distribution of PGG-paclitaxel, a novel macromolecular formulation of paclitaxel, in nu/nu mice bearing NCI-460 lung cancer xenografts. Cancer Chemother. Pharmacol..

[26] Yang D., Van S., Jiang X., Yu L. (2011). Novel free paclitaxel-loaded poly(l-gamma-glutamylglutamine)-paclitaxel nanoparticles. Int. J. Nanomed..

[27] Yang D., Van S., Shu Y., Liu X., Ge Y., Jiang X., Jin Y., Yu L. (2012). Synthesis, characterization, and *in vivo* efficacy evaluation of PGG-docetaxel conjugate for potential cancer chemotherapy. Int. J. Nanomed..

[28] Sahay G., Alakhova D.Y., Kabanov A.V. (2010). Endocytosis of nanomedicines. J. Control. Release.

[29] Wong H.L., Bendayan R., Rauth A.M., Xue H.Y., Babakhanian K., Wu X.Y. (2006). A mechanistic study of enhanced doxorubicin uptake and retention in multidrug resistant breast cancer cells using a polymer-lipid hybrid nanoparticle system. J. Pharmacol. Exp. Ther..

[30] Yang D., Liu X., Jiang X., Liu Y., Ying W., Wang H., Bai H., Taylor W.D., Wang Y., Clamme J.P. (2012). Effect of molecular weight of PGG-paclitaxel conjugates on *in vitro* and *in vivo* efficacy. J. Control. Release.

[31] Zahreddine H., Borden K.L. (2013). Mechanisms and insights into drug resistance in cancer. Front. Pharmacol..

[32] Gottesman M.M. (2002). Mechanisms of cancer drug resistance. Ann. Rev. Med..

[33] Krishna R., Mayer L.D. (2000). Multidrug resistance (MDR) in cancer. Mechanisms, reversal using modulators of MDR and the role of MDR modulators in influencing the pharmacokinetics of anticancer drugs. Eur.J. Pharm. Sci..

[34] Cheng Q., Du L., Meng L., Han S., Wei T., Wang X., Wu Y., Song X., Zhou J., Zheng S. (2016). The Promising Nanocarrier for Doxorubicin and siRNA Co-delivery by PDMAEMA-based Amphiphilic Nanomicelles. ACS Appl. Mater. Interfaces.

